# Protocol for the establishment of murine orthotopic lung tumors and the assessment of contralateral pulmonal metastasis by flow cytometry

**DOI:** 10.1016/j.xpro.2025.103793

**Published:** 2025-04-23

**Authors:** Gregor Zaun, Siyang Liu, Maximilian Webendörfer, Abubakar Wani, Diego Rodriguez, Douglas R. Green, Halime Kalkavan

**Affiliations:** 1 Department of Medical Oncology, West German Cancer Center, University Hospital Essen, University Duisburg-Essen, 45122 Essen, Germany; 2 Medical Faculty, University Duisburg-Essen, Essen, Germany; 3 Department of Immunology, St. Jude Children’s Research Hospital, Memphis, TN 38105, USA; 4 Institute for Clinical Chemistry and Clinical Pharmacology, University Hospital Bonn, Bonn, Germany; 5 German Cancer Consortium (DKTK), Partner Site University Hospital Essen, Essen, Germany; 6 National Center for Tumor Diseases (NCT) West, Campus Essen, 45122 Essen, Germany

**Keywords:** Cell Biology, Single Cell, Flow Cytometry, Cancer, Model Organisms

## Abstract

Murine orthotopic tumor models can accurately resemble cancer biology characteristics including metastasis, drug sensitivity, and remodeling of the tumor microenvironment. Here, we present a protocol for establishing murine orthotopic lung tumors and assessing contralateral pulmonary metastasis by flow cytometry. We describe steps for marking tumor cells with label retention dyes, preparing cell mixtures in Matrigel, and performing intrapulmonary injection. We then detail procedures for *ex vivo* processing of lungs followed by fluorescence-activated cell sorting (FACS) data analysis and quantification of metastatic capacity.

For complete details on the use and execution of this protocol, please refer to Kalkavan et al*.*^1^

## Highlights

• Protocol for the comparative assessment of metastatic capacity of cancer cells
• Step-by-step guide for efficient sample preparation and orthotopic lung tumor injection
• Using flow cytometry for the detection of metastatic dissemination in mice

## Abstract (editor highlights)

Publisher’s note: Undertaking any experimental protocol requires adherence to local institutional guidelines for laboratory safety and ethics.

## Graphical abstract

**Figure:**
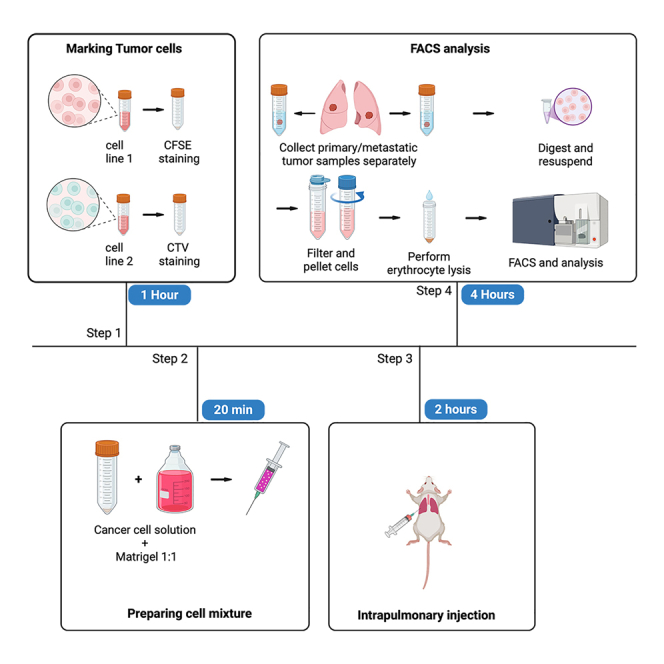

## Before you begin

The investigators should be proficient with injecting mice transcutaneously and the use of a flow cytometer.

***Note:*** This protocol can be performed without the need for label retention dyes by using fluorescent probes or use of cells that are expressing fluorescent proteins which help identifying and differentiating diverse cell lines. If that is the case, skip steps 1–16 and make cell suspensions of each cell line 1 and 2 separately, containing 5 × 10^6^ cells in 125 μL in complete medium (which suffices for n = 5 mice). And continue with step 17.

### Institutional permissions

The St. Jude Institutional Animal Care and Use Committee approved all procedures in accordance with the Guide for the Care and Use of Animals.

### Preparation of reagents

1. Prepare stock solutions of label retention dyes (e.g., CFSE and CTV)^2^ according to manufacturer’s instructions. (https://assets.thermofisher.com/TFS-Assets/LSG/manuals/MAN0002595_CellTrace_Cell_Proliferation_Kits_UG.pdf).
2. Prepare Matrigel aliquots according to manufacturer’s protocol and freeze aliquots of 150 μL at −20°C. (https://www.sigmaaldrich.com/DE/de/technical-documents/protocol/cell-culture-and-cell-culture-analysis/3d-cell-culture/corning-matrigel-basement-membrane-matrix-for-3d-culture-in-vitro).

## Key resources table

**Table:** 

REAGENT or RESOURCE	SOURCE	IDENTIFIER
**Antibodies**		

CD31 – PE Cy7 (use 1:100)	BD	Cat# 561410 RRID: AB_10612003
CD45 – APC (use 1:100)	BD	Cat# 559864; RRID: AB_398672

**Chemicals, peptides, and recombinant proteins**		

Liberase	Sigma-Aldrich	Cat# 5401119001
DNase I, grade II, from bovine pancreas	Sigma-Aldrich	Cat# 10104159001
Corning Matrigel basement membrane matrix, LDEV-free	Corning	Cat# 356234
CellTrace CFSE cell proliferation kit, for flow cytometry	Thermo Scientific	Cat# C34554
CellTrace Violet (CTV)	Thermo Scientific	Cat# C34557
RPMI 1640	Gibco (Thermo Fisher Scientific)	Cat#11875085
FBS	Gibco (Thermo Fisher Scientific)	Cat#A5256701
Penicillin-streptomycin	Gibco (Thermo Fisher Scientific)	Cat#15140122
GlutaMAX 100×	Gibco (Thermo Fisher Scientific)	Cat#35050061
ACK-lysis buffer	Gibco (Thermo Fisher Scientific)	Cat#A1049201
Sodium azide	Merck	Cat#S2002
Dulbecco’s phosphate-buffered solution (DPBS) powder without calcium and magnesium	Gibco (Thermo Fisher Scientific)	Cat#21600044
UltraPure 0.5 M EDTA, pH 8.0	Invitrogen (Thermo Fisher Scientific)	Cat#15575020

**Experimental models: Cell lines**		

*H. sapiens: PC9*	MilliporeSigma	Cat# 90071810

**Experimental models: Organisms/strains**		

NOD.Cg-Prkdcsscid Il2rgtm1Wjl/SzJ (NSG) (5- to 12-weeks-old females and males)	In-house	N/A

**Software and algorithms**		

FlowJo v.10	FlowJo, LLC	https://www.flowjo.com
GraphPad Prism 9.0	GraphPad Software	https://www.graphpad.com/scientific-software/prism/

## Materials and equipment

**Table:** Complete culture medium

Reagent	Final concentration	Amount
RPMI 1640	N/A	440 mL
FBS	10%	50 mL
Glutamax	1%	5 mL
penicillin/streptomycin	1%	5 mL
**Total**	**N/A**	**500 mL**

Store at −4°C for up to 3 months.

**Table:** FACS buffer

Reagent	Final concentration	Amount
Milli-Q filtered water	N/A	up to 1 L
FBS	1%	10 mL
EDTA 0.5 M	2.5 mM	5 mL
Sodium Azide	0.1% (w/v)	1 g
DPBS Powder	1% (w/v)	10 g
**Total**	**N/A**	**1 L**

Store at 20°C–25°C for up to 6 months.

## Step-by-step method details

### Marking tumor cells with label retention dyes

**Timing: 60 min**

***Note:*** Any additional treatments to tumor cells should be performed before the staining with label retention dyes.

***Note:*** The cell numbers and volumes below are sufficient for 5 mice.

1. Make single cell suspensions of 5 x 10^6^ cells each from two different tumor cell lines from culture, which shall be compared.
2. Centrifuge cells at 400–800 g for 5 min, discard supernatant.
3. Wash cells once in Dulbecco’s Phosphate-buffered solution (DPBS) by gently pipetting up and down with a 1 mL pipette, then top up to 10 mL DPBS.
4. Spin down cells at 400–800 g for 5 min, discard supernatant.
5. Repeat steps 3 and 4.
6. Resuspend cells in 2.5 mL DPBS each.
7. Prepare 2.5 mL of 16 μM solutions of the label retention dyes CFSE (stock solution 5 mM) and CTV (stock solution 5 mM) in DPBS.
8. Quickly mix the 2.5 mL CFSE into cell line 1 and the CTV into cell line 2.
9. Gently mix by turning the Falcon tubes up and down approx. 10 times.
10. Incubate at 37°C for 15 min.
11. Add 8 mL of complete culture medium and mix by turning up and down approx. 5 times.
12. Incubate at 37°C for 5 min.
13. Pellet the cells by spinning down at 400–800 g for 5 min, discard supernatant.
14. Resuspend cells in 8 mL of complete culture medium.
15. Pellet the cells by spinning down at 400–800 g for 5 min, discard supernatant.
16. Resuspend cells in 8 mL of complete culture medium.

***Note:*** For FACS controls take 10 μL of each stained cell line separately and add each into 1 well of 6 well-plate with 2 mL of complete medium. Keep in incubator until FACS analysis.

17. Combine both tubes of stained cells in a 50 mL Falcon tube.
18. Pellet the cells by centrifugation at 400–800 g for 5 min, discard supernatant.
19. Resuspend cells in 120 μL complete culture medium for a final volume of approximately 125 μL containing 10 x 10^6^ labeled tumor cells.

***Note:*** This cell number was feasible and sufficient in our model with flow cytometric readout for comparing metastatic capacity between different cell lines. Cell count variability has not been tested. If desired, researchers can modify cell count numbers e.g. to use our tumor-injection model for different readouts like tumor development and microenvironment e.g. by CT scan or IHC.

### Preparing cell mixtures in Matrigel

**Timing: 20 min**

20. Thaw 150 μL of Matrigel aliquot on ice.
21. Precool five 1-mL syringes with 30-gauge hypodermic needles on ice.
22. Mix 125 μL cancer cell solution 1:1 into Matrigel and keep on ice (plan 50 μL/inj.).
23. Draw up each 1-mL syringe with the tumor cell / Matrigel mixture to obtain with single volume of 50 μL (2 × 10^6^ total cells per mouse).

### Intrapulmonary injection

**Timing: approximately 2 h**

**Figure 1:**
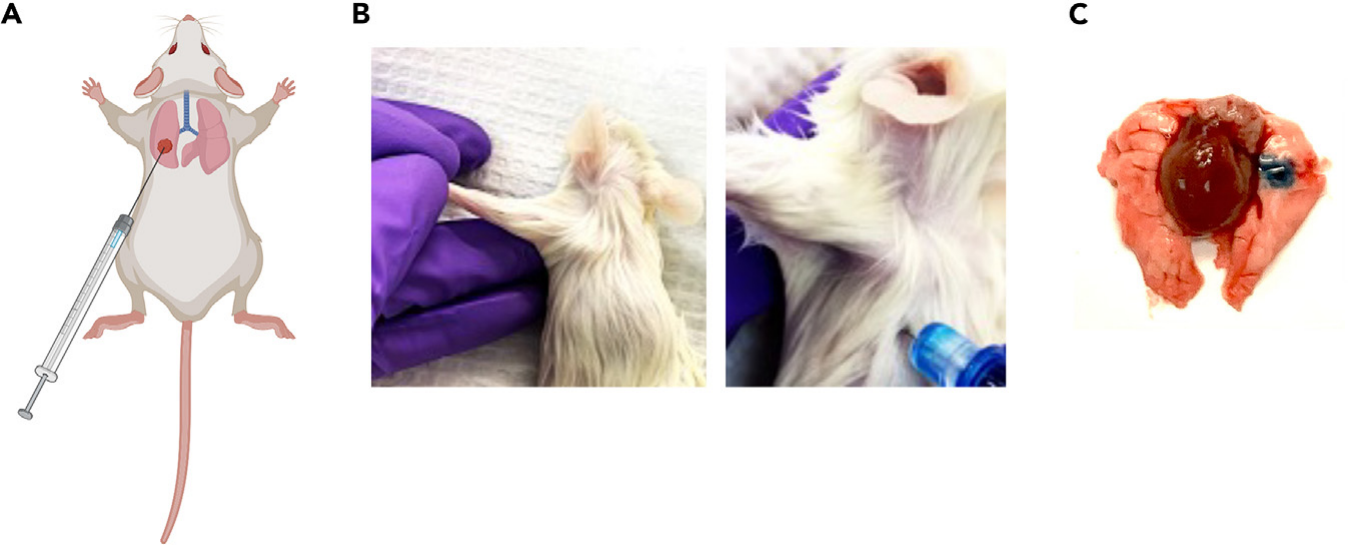
Transthoracic intrapulmonary injection (A) Schematic dorsal view of transthoracic intrapulmonary injection. (B) Photographs showing localization of injection site below the scapula, while slightly lifting the ipsilateral arm. (C) Photograph of the resected and collapsed lungs with heart in the middle, after intrapulmonary injection of tumor cells.
28. After injection turn mice to the left lateral decubitus position and observe for 15 min.

***Note:*** Minor, self-limiting nose bleeding can occur occasionally after intrapulmonary injection. No distress or injection-related complications have been observed.

***Note:*** This non-surgical approach is comparable to the published surgical procedure for intrapulmonary injection into the left lung.^3^^,^^4^ However, removing the need for surgery makes this assay less burdensome for animals by shortening procedure time and avoiding cutaneous wounds. Moreover, transthoracic non-surgical approaches have been described for the right lung, where immunohistochemical (IHC) approaches and CT scans have been used as readouts.^5^^,^^6^ Our presented protocol uses a flow cytometric approach. This enables the distinction of contemporarily injected different cell lines within the same tumor cell – Matrigel - mixture (as opposed to CT scan) and increases sensitivity by the detection of disseminated single tumor cells in the whole contralateral lung, which would be limited even in an IHC approach, since only tissue sections could be evaluated.

24. Anesthetize mice with isoflurane by standard local protocol (e.g., induced in a chamber with 2–3% Isoflurane in oxygen for approximately 5 min).
25. Place mouse in right lateral decubitus position.
26. Perform local skin disinfection of the left posterior thorax (e.g., apply Chlorhexidine Solution 2% or 70% isopropyl alcohol, mice can be replaced into chamber for a contact time of 5 min).
27. Execute transdermal intrapulmonary injections at the posterior medial line juxta below the inferior angle of the scapula, orthogonal through the intercostal space, 5–7 mm into the thorax (Figure 1). You can slightly lift the left arm of the mouse to expose the scapula, while pushing gently the thorax upwards with the 4^th^ and 5^th^ digits of your left hand.

### *Ex vivo* processing of lungs for FACS analysis

**Timing: approximately 4 h**

***Note:*** For label retention dyes perform resection of lungs within 1–7 days after injection.

29. Prepare 10 × 50 mL Falcon tubes with 10 mL of 1× DPBS and label them for mouse 1–5, left and right lung separately.
30. Sacrifice the mice according to standard local facility protocol.
31. Resect left lung (primary tumor injection site) and right lung (metastatic side) of animals and place in separate Falcon tubes.
32. Place organs in 6 well plates (one whole lung per well, meaning left and right lungs always kept in separate wells).
33. Mince tissues using scissors and digest in 1 mL trypsin supplemented with 100 μg/mL Liberase (Sigma-Aldrich) and 200 μg/mL Deoxyribonuclease I (Sigma-Aldrich) for 30 min incubation at 37°C 5% CO_2_.
34. Resuspend organs in DMEM containing 5% FCS and pipette up-and down 3 times.
35. Pass the organ suspension through a 40 μM cell strainer into a 50 mL Falcon tube.
36. Pellet the cells by spinning down at 400–800 g for 5 min, discard supernatant.

***Optional:*** Stain cells for surface markers CD45-APC and CD31-PECy7 for 20 min at 4°C (use antibodies in 1:100 dilution).

37. Perform erythrocyte lysis with ACK buffer at room temperature for 2–5 min according to manufacturer’s protocol.
38. Wash cells in FACS Buffer before FACS analysis.
39. Pellet the cells by spinning down at 400–800 g for 5 min, discard supernatant.
40. Resuspend cells in 400 μL FACS Buffer.
41. Pass the cells through a cell strainer into a FACS tube to ensure a single cell suspension.
42. Trypsinize single color stained cells from 6 well plate and unstained cells from cell culture, and resuspend in 300 μL FACS buffer (to be used later as single-color FACS controls).
43. Perform flow cytometry. This protocol has been successfully executed using an AURORA 3 L and 4 L. However, it will be applicable to other Cytometers.

### FACS data analysis and quantification of metastatic capacity

**Timing: 4+ h**

**Figure 2:**
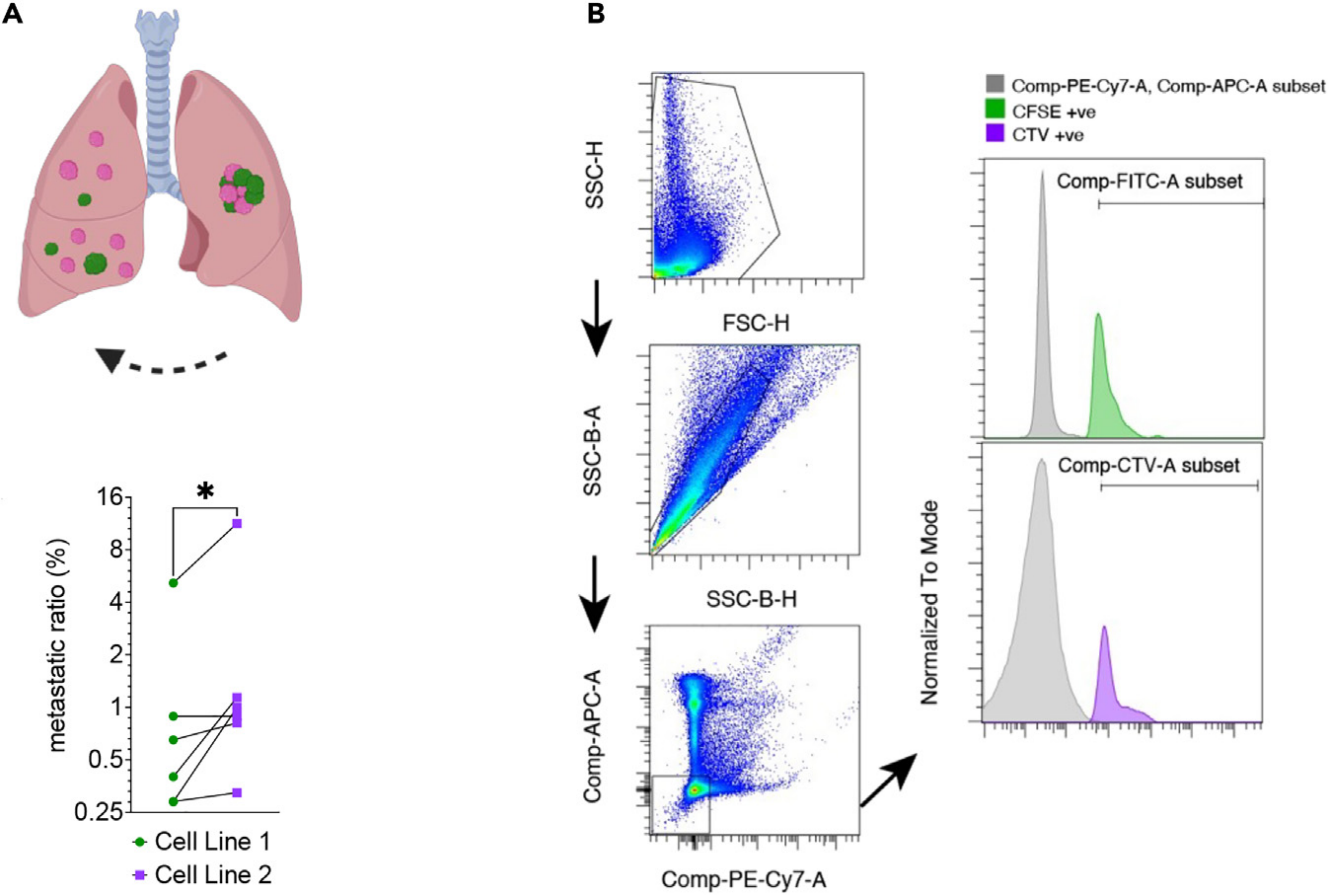
Analysis of contralateral pulmonary metastasis (A) Scheme for transthoracic contralateral pulmonal metastasis of two differently labeled cancer cell lines. (B) Gating strategy. In this overlay, histograms contain total APC and PE-Cy-7 negative group, and its subpopulations of selected FITC (CFSE^+^ cell line 1) or CTV (cell line 2) positive cells – for demonstration of subpopulation separation. (C) Ratio of right pulmonal (metastatic site) versus left pulmonal (injection site) cancer cells detected by flow cytometry, paired t-test, *p*-value ∗ <0.05.

This step describes the gating strategy and quantification of metastatic capacity. The gating strategy is shown in Figure 2.

44. Run each control, starting with unstained sample to optimize the voltage, gating, and compensation matrix.

45. Use the unstained sample to identify sample and single cell populations.
  ***Optional:*** Use CD45(-APC) and CD31(PE-Cy7) to exclude lymphocytes and endothelial cells (see troubleshootingproblem 3).
  a. Use CFSE or CTV respectively vs. SSC to gate for Cancer Cells (see troubleshooting problem 3).
  b. Quantify metastatic capacity by using the ratio between the detected number of CFSE (or CTV respectively) positive cells in the contralateral lung divided by the cell number from the injected lung.

## Expected outcomes

Upon successful completion of this protocol diverse cancer cells should be detected in lung tissue and a metastatic ratio can be quantified. While in theory the ratio can reach numbers from 0–100%, ratios between 0–10% have been assessed routinely for different human lung cancer cells.^1^

## Limitations

The quality of this assay’s results strongly depends on various factors like the viability of cancer cells injected, the proper handling of Matrigel, the skilled injection of tumor cells into the lung and the processing of lungs after harvesting organs. The generation of single cell suspensions after mincing of lungs, is a crucial step where the incubation with trypsin, liberase and DNase is important to free single cancer cells from adherent tissue, but which can also be a source of cell degradation. If label retention dyes are used, then the assay must be finalized within a few days (depending on cancer cell doubling time). A prolonged incubation time might lead to a decrease in overall signal and will limit cancer cell detection. While the use of fluorescent intracellular proteins can overcome this problem, their expression can vary within a cell culture. This must be normalized e.g., via previous sorting.

## Troubleshooting

### Problem 1

Label retention dye and antibody solutions might not be optimal for all cancer cells and mouse models.

### Potential solution

Titration and optimization are recommended for all antibodies and dyes using best practices for flow cytometry.^7^

### Problem 2

Cancer cell retrieval after lung processing might be insufficient.

### Potential solution

A crucial step for the release of single cancer cells from tissues is the incubation with trypsin, liberase and DNase. Optimization of incubation time is crucial for a successful assay.

### Problem 3

Gating and detection of cancer cells in contralateral lungs is unsuccessful.

### Potential solution

Since low numbers of cancer cells will be metastasized into contralateral lungs, millions of single cells from lung tissue must be acquired by FACS (e.g., at least 1 × 10^6^ events per lung). This makes gating challenging. Aliquot of controls with lung cells with or without manually added original CFSE or CTV positive cells, helps acquire a small number of cells with FACS (e.g., 10000 cells) and to define gates before whole sample assessments. Since cancer cell lines will be injected simultaneously and ratios between lungs are used, any potential over- or underestimation due to analytic challenges or due to technical reasons would affect all test groups in the same way and will therefore not impact pairwise statistical comparison.

### Problem 4

Uncertainty about injection accuracy and reproducibility.

### Potential solution

As for any other invasive *in vivo* technique (like for instance i.v. or i.p. injections), practice of proper handling is crucial. Scientists that are less experienced with mouse invasive techniques, should get guidance from an experienced researcher. In general, the comparative nature (ratio between two lungs, contemporal injection of cell lines) minimizes technical errors. FACS analysis of the injected lung containing labeled cancer cell lines will serve as a positive control for accuracy. If additional controls are desired untreated lungs can be used as complementary negative controls. Moreover - although this protocol is focusing on FACS-based analysis - a combination with *in vivo* imaging approaches is of course possible, if further validation is desired. This might include small animal CT scans or intravital imaging (IVIS) e.g., of luciferase producing cancer cells.

## Resource availability

### Lead contact

Requests for further information should be directed to and will be fulfilled by the lead contact, Dr. Halime Kalkavan, halime.kalkavan@uk-essen.de.

### Technical contact

Requests for further information should be directed to and will be fulfilled by the lead contact and first author, Dr. Halime Kalkavan, halime.kalkavan@uk-essen.de, and Dr. Gregor Zaun, gregor.zaun@uk-essen.de.

### Materials availability

This paper did not generate any new reagents. All materials used can be purchased from the manufacturers.

### Data and code availability

This paper did not generate any new datasets or code.

## Acknowledgments

This work was supported by grants from the 10.13039/501100001659German Research Foundation (DFG, KA 4830/1-1), the Advanced Clinician Scientist program UMEA^2^ (Medical Faculty, University of Duisburg-Essen), the 10.13039/501100002347Federal Ministry of Education and Research 01EO2104 (BMBF), and 10.13039/501100005972German Cancer Aid (Max-Eder Junior Research Group Program, DKH 70115382) to H.K.; by 10.13039/100012524ALSAC (SJCRH); and by the 10.13039/100000054US National Cancer Institute grant R35 CA231620 to D.R.G. The graphical abstract was generated with BioRender.

## Author contributions

H.K. led the project. H.K. and S.L. optimized the protocol and performed experiments. S.L., G.Z., and M.W. performed analysis used in the generation of figures. H.K., D.R.G., and A.W. wrote the manuscript. All authors were involved in data interpretation and review of this manuscript.

## Declaration of interests

The authors declare no competing interests.
